# Comparison of Intravenous Dexmedetomidine Versus Dexmedetomidine-Dexamethasone Combination for Preventing Postoperative Nausea and Vomiting in Adult Patients After Abdominal Surgeries

**DOI:** 10.7759/cureus.65913

**Published:** 2024-07-31

**Authors:** Tanmay Yadav, Meenakshi Kumar, Krishika Verma

**Affiliations:** 1 Anesthesiology and Critical Care, Vardhman Mahavir Medical College and Safdarjung Hospital, Delhi, IND; 2 Anesthesiology, Critical Care and Pain Medicine, Vardhman Mahavir Medical College and Safdarjung Hospital, Delhi, IND

**Keywords:** prospective randomized comparative research, preoperative, adult, intravenous dexmedetomidine, abdominal surgeries, ponv

## Abstract

Introduction: Postoperative nausea and vomiting (PONV) is a common problem following general anesthesia and is one of the most unpleasant side effects that affects the patient after surgery and is the worst memory of the hospital stay. The present prospective randomized comparative study was designed to compare the effect of intravenous dexmedetomidine with dexmedetomidine-dexamethasone combination for preventing PONV following abdominal surgeries in adult patients and evaluating their sedative and analgesic effects.

Methodology: A total of 75 patients (aged 18-65 years) were assigned to undergo this comparative study via block randomization using a sealed envelope system. They were divided into three groups of 25 each: group A (control) received normal saline, group B received dexmedetomidine, and group C received a combination of dexmedetomidine with dexamethasone over 10 minutes after inducing general anesthesia before skin incision.

The primary outcome was to assess PONV, where nausea was assessed by the numerical rating scale and vomiting by the number of gastric content expulsions. The secondary outcome, which is postoperative sedation and pain, was assessed by the Ramsay Sedation Score and Visual Analog Score, respectively, for 24 hours postoperatively.

Result: During the first 24 hours after surgery, the incidence of PONV was similar in both dexmedetomidine and combination groups but lower than the control group. Postoperative sedation and analgesia were both statistically and clinically adequate and similar in dexmedetomidine and combination groups. No major side effects requiring pharmacological intervention were reported.

Conclusion: Dexmedetomidine alone is as effective as its combination with dexamethasone in preventing PONV in adult patients following abdominal surgeries.

## Introduction

PONV is a highly undesirable event occurring in patients. Without any prophylactic management, the incidence of PONV can rise to 80% in patients with predisposing factors, like previous history of PONV, motion sickness, and female gender [1]. Other risk factors include increased intra-abdominal pressure, reduced gastrointestinal motility, general anesthesia, use of volatile anesthetics, nitrous oxide gas, and type of surgery (cholecystectomy and laparoscopic and gynecological procedures).

A number of pharmacological agents have been tried for PONV prophylaxis, like 5-HT3 receptor antagonists, which include ondansetron, granisetron, amisulpride, and droperidol. When applied before surgery or the night before, these agents are effective for PONV prophylaxis for up to 24 hours postoperatively [2,3].

Dexmedetomidine, a highly selective α2 adrenoceptor agonist with sedative and analgesic properties, has been shown to reduce the frequency and severity of PONV in highly susceptible patients [4-7]. Dexamethasone is a long-acting glucocorticoid [8] known to be as effective as ondansetron. When used as an adjunct to another antiemetic, it has been shown to significantly increase the antiemetic efficacy compared to the primary drug alone [9]. Some studies have shown that a combination of dexmedetomidine and dexamethasone reduces PONV [10-16], while some studies have evidence against it [7,17].

Our study aimed to determine whether dexmedetomidine alone can be effectively used to prevent PONV as opposed to a combination of dexmedetomidine and dexamethasone in patients undergoing abdominal surgery under general anesthesia.

## Materials and methods

This prospective randomized comparative research was conducted in the Department of Anesthesia and Intensive Care, Vardhman Mahavir Medical College and Safdarjung Hospital, New Delhi, over a period of 18 months after an Institutional Ethics Committee approval. After acquiring written informed consent, patients aged 18-65 years, of either gender, of American Society of Anesthesiologists (ASA) physical statuses I and II, receiving general anesthesia for abdominal surgeries, were included in the study. In contrast, patients with a previous history of PONV, abdominal surgery, cardiovascular or cerebrovascular comorbidities, diabetes mellitus, history of treatment with antiemetics or glucocorticoids, and body mass index (BMI) >30 kg/m^2^ were excluded from the study.

The total sample size taken was 75 (25 per group), and it was calculated using the following formula:

SS1 ≥ [{pc × (1 - pc) + pe × (1 - pe)} × 2(Zα + Zβ)] / 2(pc-pe) N ≥ SS / [1 + {(SS−1)/Pop}].

Here, pc indicates the incidence of PONV in one group, pe is the incidence of PONV in another group, Zα is the value of Z at a two-sided α error of 5%, and Zβ is the value of Z at a power of 80%.

The calculations are as follows:

1. Incidence of PONV in the control group and dexmedetomidine group: SS1 ≥ [{0.7 × (1 - 0.7) + 0.2 × (1 - 0.2)} × 2(1.96 + 0.84)] / 2(0.7 - 0.2) ≥ 11.60 = 12 (approximately).

2. Incidence of PONV in the control group and dexmedetomidine-dexamethasone group:

SS1 ≥ [{0.7 × (1 - 0.7) + 0.12 × (1 - 0.12)} × 2(1.96 + 0.84)] / 2(0.7 - 0.12) ≥ 7.36 = 8 (approximately). 3. Incidence of PONV in the dexmedetomidine group and dexmedetomidine-dexamethasone group: SS1 ≥ [{0.2 × (1 - 0.2) + 0.12 × (1 - 0.12)} × 2(1.96 + 0.84)] / 2(0.2 - 0.12) ≥ 325.36 = 326 (approximately).

SS = 3 × SS1 = 3 × 326 = 978

The finite population correction factor is calculated using the following formula: N ≥ 978 / [1 + {(978 - 1)/80}] ≥ 74.02 = 75 (approximately).

The sealed envelope system was utilized for block randomization, and patients were divided into three groups: group A represents the control group (n = 25): this group received normal saline (10 mL); group B: dexmedetomidine group (n = 25): this group received dexmedetomidine (0.5 μg/kg) diluted to 10 mL with normal saline; group C: combination group (n = 25): this group received dexmedetomidine-dexamethasone combination (dexmedetomidine 0.5 μg/kg + dexamethasone 4 mg) diluted to 10 mL with normal saline.

All patients underwent a detailed preanesthetic evaluation. The nature of the study was explained to the patients, and informed consent was obtained from all patients willing to participate in the study. The patient was assigned randomly to any of the three groups. Preoperative HR, blood pressure (BP), SpO_2_, and blood sugars were noted.

The patient received general anesthesia as per standard protocol. The “standard guidelines” here refer to our institutional protocol for induction of general anesthesia, which is balanced anesthesia and includes opioids as premedication, intravenous induction agents, and muscle relaxants. Following preoxygenation for three minutes, anesthesia was induced with fentanyl (1-2 µg/kg), propofol (1-2.5 mg/kg), and vecuronium bromide (0.08-0.1 mg/kg). After endotracheal intubation, anesthesia was maintained with 0.5%-1% isoflurane and 50% nitrous oxide with oxygen. Mechanical ventilation was adjusted to maintain an end-tidal carbon dioxide tension at 35-45 mmHg.

Patients in each of the respective groups were given the study drug/s by resident doctors who did not participate in the study before the skin incision over a period of 10 minutes.

Supplemental doses of muscle relaxants and opioids were administered as and when required. Intraoperative vital signs were monitored. At the end of the surgery, anesthesia was reversed using reversal agents, and the patient was extubated. Any PONV at the end of surgery was managed with ondansetron (0.15 mg/kg). Postoperatively, blood sugar was recorded.

All patients were monitored for bradycardia and hypotension, and heart rate (HR) <45 bpm was managed using atropine. Hypotension (either systolic blood pressure (SBP) <20% of baseline or mean arterial pressure <65 mmHg) was managed using fluids and, if required, mephentermine.

All patients were monitored for postoperative nausea, vomiting, sedation, pain, and blood sugar at 1, 3, 6, 9, 12, and 24 hours postoperatively.

Postoperative nausea was assessed using an 11-point numerical rating scale (NRS), with 0 being no symptoms and 10 being the worst symptoms imaginable. NRS >5 was managed with ondansetron (0.15 mg/kg). Postoperative vomiting was assessed as the frequency of expulsion of any gastric contents in the first 24 hours following surgery.

Postoperative sedation was assessed using the Ramsay sedation scale (RSS) score: 1: agitated, anxious, or restless; 2: oriented, cooperative, and tranquil; 3: responsive to verbal commands only; 4: asleep, brisk response to a loud auditory stimulus or a light glabella tap; 5: sluggish response to a loud auditory stimulus or a light glabella tap; 6: no response to a loud auditory stimulus or a light glabella tap. RSS >4 was considered as oversedation. Postoperative pain was assessed using a visual analog scale (VAS). VAS >4 was managed with 0.5-1 µg/kg of fentanyl.

Statistical analysis

The data were entered into a Microsoft Excel spreadsheet, and analysis was performed using the Statistical Package for the Social Sciences version 25.0.

Categorical variables were presented in number and percentage (%), and continuous variables were presented as mean± SD. The normality of data was tested using the Kolmogorov-Smirnov test. If the normality was rejected, a nonparametric test was used.

The statistical tests applied are as follows: (1) quantitative variables were compared using analysis of variance or the Kruskal-Wallis test (when the datasets were not normally distributed) to test for significance between the three groups, and (2) Tukey HSD test or Dunn’s test (when the datasets were not normally distributed) was then used to find out the pair of groups between which significance existed. A p value of <0.05 was considered statistically significant.

## Results

Parameters like age, BMI, sex, and ASA grading were compared between groups, and the values were statistically nonsignificant. The mean duration of surgery in group A was 108.76 minutes, in group B was 111.60 minutes, and in group C was 110.68 minutes, with no statistically significant difference between the three groups (p = 0.877).

Vital parameters

**Figure 1:**
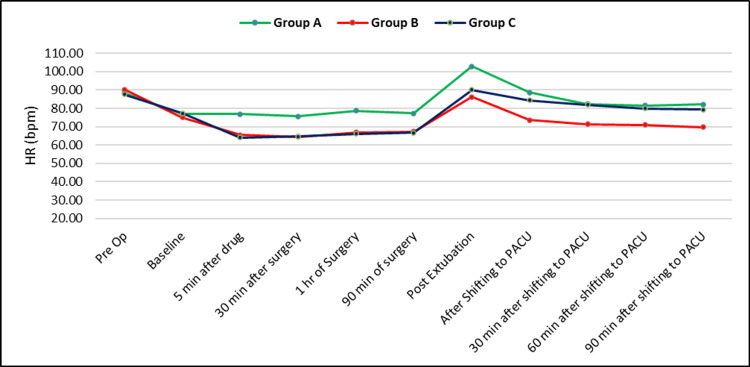
The trend of HR in the study groups HR: heart rate; PACU: postanesthesia care unit

At baseline, the HR was not different between groups. However, five minutes after the drugs were administered, there was a difference in HR, which was statistically significant between groups A and C and groups A and B (Figure 1).

**Figure 2:**
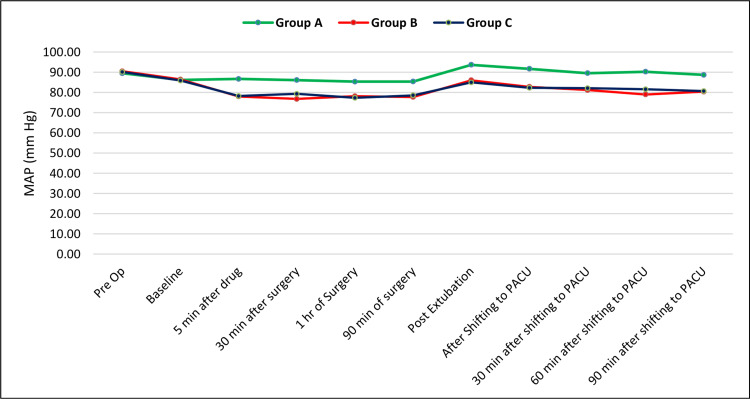
The trend of MAP in the study groups MAP: mean arterial pressure; PACU: postanesthesia care unit

There is a statistically significant difference in the mean arterial BP between groups A and B, and groups A and C from five minutes after drug administration until 90 minutes after shifting to the postanesthesia care unit (p < 0.05), with a higher mean SBP in group A than in groups B and C at all these time points (Figure 2).

Postoperative Nausea and Vomiting

There is a statistically significant difference in the mean NRS score between groups A and B, and groups A and C from the end of surgery until 12 hours after surgery (p < 0.05), with a lower mean NRS score in groups B and C than in group A at all these time points except at 6 and 12 hours after surgery. The reason was that pharmacological intervention was given postextubation in the control group (Table 1).

**Table 1:** Comparison of nausea (NRS score) between groups ^a^Group A vs. group B ^b^Group A vs. group C NRS: numerical rating scale

Nausea (NRS score)	Group A	Group B	Group C	p value
	Mean ± SD	Mean ± SD	Mean ± SD	
At the end of the surgery	4.56 ± 3.1	1.92 ± 1.26	2.16 ± 1.52	0.007^a,b^
1 hour after surgery	3.24 ± 2.11	0.64 ± 0.49	0.6 ± 0.65	<0.001^a,b^
3 hours after surgery	2.04 ± 1.54	0.84 ± 0.85	0.92 ± 0.7	0.005^a,b^
6 hours after surgery	0.2 ± 0.41	1.64 ± 2.2	1.4 ± 2.24	0.002^a,b^
9 hours after surgery	4.84 ± 1.75	2.44 ± 1.5	2.16 ± 1.28	<0.001^a,b^
12 hours after surgery	1.44 ± 0.96	3.64 ± 1.6	3.28 ± 1.9	<0.001^a,b^
24 hours after surgery	4.12 ± 2.47	3.48 ± 2.5	3.4 ± 2.4	0.55

There is a statistically significant difference in the mean number of incidences of vomiting in both the study groups when compared with the control group, between groups A and B, and groups A and C at the end of the surgery, and at 1, 6, and 9 hours after surgery (p < 0.05). The mean number of incidences of vomiting is higher in group A at the end of surgery and at 1 and 6 hours after surgery and lower in group A at 9 hours after surgery. There is no statistically significant difference in the mean number of incidences of vomiting between groups B and C from the end of surgery until 24 hours postoperatively (Table 2).

**Table 2:** Comparison of postoperative vomiting between groups ^a^Group A vs. group B ^b^Group A vs. group C

Vomiting (number of incidences)	Group A	Group B	Group C	p value
	Mean ± SD	Mean ± SD	Mean ± SD	
At the end of the surgery	0.44 ± 0.51	0.04 ± 0.2	0	<0.001^a,b^
1 hour after surgery	0.24 ± 0.44	0	0	0.002^a,b^
3 hours after surgery	0	0.04 ± 0.2	0.16 ± 0.37	0.064
6 hours after surgery	0.2 ± 0.41	0	0	0.005^a,b^
9 hours after surgery	0.08 ± 0.28	0.48 ± 0.51	0.4 ± 0.5	0.006^a,b^
12 hours after surgery	0.12 ± 0.33	0.16 ± 0.37	0.4 ± 0.5	0.040^b^
24 hours after surgery	0.08 ± 0.28	0.12 ± 0.33	0.12 ± 0.33	0.871

Postoperative Sedation

There is a statistically significant difference in the mean RSS score between groups A and B at the end of surgery and 9 hours after surgery (p < 0.05) and higher in group B. There is a statistically significant difference in the mean RSS score between groups A and C at the end of surgery and at 6 and 9 hours after surgery (p < 0.05), higher in group C at the end of surgery and at 9 hours after surgery, and higher in group A at 6 hours after surgery. There is a statistically significant difference in the mean RSS score between groups B and C at six hours postoperatively (higher in group B). Group B shows a mostly stable trend of the mean RSS score from the end of surgery until six hours after surgery, followed by an insignificant fall at nine hours after surgery, followed by a further stable trend (Table 3).

**Table 3:** Comparison of sedation (RSS score) between groups ^a^Group A vs. group B ^b^Group A vs. group C ^c^Group B vs. group C RSS: Ramsay sedation scale

Sedation (RSS score)	Group A	Group B	Group C	P value
	Mean ± SD	Mean ± SD	Mean ± SD	
At the end of the surgery	1.56 ± 0.51	2.00 ± 0.00	1.84 ± 0.37	<0.001^a,b^
1 hour after surgery	1.88 ± 0.33	2.00 ± 0.00	1.88 ± 0.33	0.2
3 hours after surgery	2.00 ± 0.00	2.00 ± 0.00	2.00 ± 0.00	1
6 hours after surgery	2.00 ± 0.00	2.00 ± 0.00	1.84 ± 0.37	0.015^b,c^
9 hours after surgery	1.56 ± 0.51	1.88 ± 0.33	1.92 ± 0.28	0.003^a,b^
12 hours after surgery	2.00 ± 0.00	1.92 ± 0.28	1.80 ± 0.41	0.052
24 hours after surgery	2.00 ± 0.00	1.88 ± 0.33	1.88 ± 0.33	0.2

Postoperative Pain

There is a statistically significant difference in the mean VAS score between groups A and B (p < 0.001) and between groups A and C (p < 0.001) at all time points assessed (Table 4).

**Table 4:** Comparison of pain (VAS score) between groups ^a^Group A vs. group B ^b^Group A vs. group C VAS: visual analog scale

Pain (VAS score)	Group A	Group B	Group C	p value
	Mean ± SD	Mean ± SD	Mean ± SD	
At the end of the surgery	4.84 ± 0.85	1.36 ± 1.11	0.92 ± 1.19	<0.001^a,b^
1 hour after surgery	3.28 ± 0.46	0.64 ± 0.81	0.44 ± 0.82	<0.001^a,b^
3 hours after surgery	2.28 ± 0.46	0.64 ± 0.81	0.44 ± 0.82	<0.001^a,b^
6 hours after surgery	3.84 ± 0.85	1.36 ± 1.11	0.44 ± 0.82	<0.001^a,b^
9 hours after surgery	2.84 ± 0.85	1.36 ± 1.11	0.92 ± 1.19	<0.001^a,b^
12 hours after surgery	2.84 ± 0.85	0.64 ± 0.81	0.44 ± 0.82	<0.001^a,b^
24 hours after surgery	4.64 ± 1.15	0.64 ± 0.81	0.44 ± 0.82	<0.001^a,b^

Side Effects

A total of 17 participants had intraoperative bradycardia (three in group A and seven each in groups B and C), with no statistically significant difference in the incidence of intraoperative bradycardia between the study groups (p = 0.296). Out of the 17 participants who had intraoperative bradycardia, three participants (one in each group) required atropine to manage bradycardia. A total of four participants had postoperative bradycardia until the time HR was monitored (zero in group A and two each in groups B and C), with no statistically significant difference in the incidence of postoperative bradycardia between the study groups (p = 0.348). None of the participants who had postoperative bradycardia required any intervention to manage bradycardia (Table 5).

**Table 5:** Comparison of bradycardia between groups

Bradycardia		Group A	Group B	Group C	p value
		N	N	N	
Bradycardia intra-op	No	22 (88%)	18 (72%)	18 (72%)	0.296
	Yes	3 (12%)	7 (28%)	7 (28%)	
Bradycardia post-op	No	25 (100%)	23 (92%)	23 (92%)	0.348
	Yes	0 (0%)	2 (8%)	2 (8%)	
Arrhythmia intra-op	No	25 (100%)	25 (100%)	25 (100%)	NS
Arrhythmia post-op	No	25 (100%)	25 (100%)	25 (100%)	
Hypotension intra-op	No	23 (92%)	20 (80%)	21 (84%)	0.474
	Yes	2 (8%)	5 (20%)	4 (16%)	
Hypotension post-op	No	25 (100%)	23 (92%)	23 (92%)	0.348
	Yes	0 (0%)	2 (8%)	2 (8%)	

No patient in our study had intraoperative or postoperative arrhythmia until the time ECG was monitored. A total of 11 participants had intraoperative hypotension (two in group A, five in group B, and four in group C), with no statistically significant difference in the incidence of intraoperative hypotension between the study groups (p = 0.474).

Except at the end of the surgery, the mean blood sugar level was significantly higher in group C compared to groups A and B until 24 hours after surgery (p < 0.001). There is no statistically significant difference in the mean blood sugar level between groups A and B postoperatively. No patient in our study had postoperative blood sugar levels significant enough to warrant intervention (Table 6).

**Table 6:** Comparison of blood sugar (mg %) between groups ^a^Group A vs. group C ^b^Group B vs. group C

Blood sugar (mg %)	Group A	Group B	Group C	p value
	Mean ± SD	Mean ± SD	Mean ± SD	
At the end of the surgery	124.12 ± 14.73	124.24 ± 12.48	130.80 ± 13.18	0.142
1 hour after surgery	118.36 ± 11.99	118.28 ± 10.86	143.24 ± 13.05	<0.001^a,b^
3 hours after surgery	120.40± 11.45	120.00 ± 10.45	149.44 ± 13.33	<0.001^a,b^
6 hours after surgery	122.72 ± 12.05	122.00 ± 11.36	155.04 ± 13.28	<0.001^a,b^
9 hours after surgery	123.40 ±12.41	121.44 ± 11.56	159.64 ± 13.71	<0.001^a,b^
12 hours after surgery	122.60 ± 12.37	119.44 ± 11.97	160.72 ± 12.95	<0.001^a,b^
24 hours after surgery	120.32 ± 11.86	116.76 ± 12.26	159.80 ± 13.36	<0.001^a,b^

## Discussion

Nausea and vomiting are among the most common postoperative complaints, which cause great distress to patients and are often the worst memories of their hospital stay [18]. Abdominal surgeries are responsible for more emesis due to manipulation of the gut, which results in vagal and splanchnic afferent discharge leading to PONV. Consequences of prolonged PONV include physical (stress on sutures and wound dehiscence, bleeding from the surgical site, gastric content regurgitation, and aspiration), physiological (increase or decrease in heart rate and BP), logistical (delayed discharge from hospital and unanticipated repeat admissions), and economic (increased cost of care). Better anesthetic techniques, identification of precipitating factors, use of a new generation of antiemetics, and improvement in operative techniques reduced the incidence and severity of PONV over the last 10 years. Despite these changes, there is still an unacceptable frequency of PONV, with incidents up to 85% reported in some studies [19].

In our study, the mean NRS score for nausea and PONV was almost equal in the dexmedetomidine group and dexmedetomidine-dexamethasone at most of the time points assessed. However, their differences are statistically insignificant during the first 24 hours. Both the groups show statistically significant differences in mean NRS score compared to the control group until 12 hours postoperatively. For PONV, the control group showed an erratic trend. It was because, in the preceding hours, nausea was severe enough to lead to rescue antiemetic administration, leading to a lesser number of incidences of vomiting. Initially, after surgery, the vomiting was higher in the control group, but due to antiemetics, it decreased subsequently compared to other groups and was statistically significant.

Similar findings were reported by Kwak et al. [10], who compared the antiemetic efficacy of dexmedetomidine versus dexmedetomidine-dexamethasone combination in patients undergoing breast surgery, and Bakri et al. [11], who compared the effect of a single dose of dexmedetomidine(1 μg/kg) to dexamethasone(8 mg) in reducing PONV following laparoscopic cholecystectomy. A similar finding was reported by Abdelmageed et al. [12], who reported that PONV was significantly reduced in the dexmedetomidine group during the first 24 hours postoperatively. They attributed their finding to the reduction of postoperative morphine consumption in the dexmedetomidine group. Moreover, Goksu et al. [13] used dexmedetomidine for sedation during functional endoscopic sinus surgery under local anesthesia. They reported a significantly lower incidence of PONV in the dexmedetomidine group, compared to a placebo group, without adverse effects. Kim et al. [14] found that the overall incidence of PONV during the 24 hours after surgery showed a trend toward a lower incidence in the dexmedetomidine group, but it did not reach statistical significance. However, they reported that dexmedetomidine significantly reduced the incidence of severe PONV during the first 24 hours after surgery. Possible explanations for this may be related to the reduced consumption of intraoperative and postoperative opioids and inhaled anesthetics [15]. In 2017, Jin et al. conducted a review to evaluate the role of dexmedetomidine in preventing nausea and vomiting in general anesthesia. The authors suggested that it could reduce nausea and vomiting if the dexmedetomidine side effects could be reduced [16], and their results were in line with our study. This is contrary to the study done by Rekei et al. [7], who compared the prophylactic effect of dexamethasone (4 mg), dexmedetomidine (25 μg), and their combination on PONV. The reason could be the difference in the strength of the dose. Kleif et al. [17] also conducted a study aimed to define the effect of preoperative dexamethasone on PONV. Nausea and vomiting were assessed on the first postoperative day, and 120 patients were enrolled. They stated that dexamethasone did not reduce nausea and vomiting.

We found a relatively stable trend of mean RSS score in the dexmedetomidine and combination groups compared to the control group. The mean RSS score was mostly higher in the dexmedetomidine group than in the combination group (p value 0.015-0.001 at different time intervals), suggesting that dexmedetomidine has the added benefit of providing adequate postoperative sedation compared to placebo. None of our study participants reported oversedation (RSS >4). Similar results were reported by Bakri et al. [11], who reported that higher RSS scores were found in the dexmedetomidine group than in the dexamethasone group.

Our study shows significantly better mean VAS scores for postoperative pain in participants receiving dexmedetomidine compared to placebo, whether alone or in combination with dexamethasone. Although mean VAS scores are lower in participants receiving dexmedetomidine-dexamethasone combination than in the dexmedetomidine alone group, their differences are statistically insignificant at all time points assessed (p value 0.001 at different time intervals). These findings are consistent with the study by Kwak et al. [10], who reported that pain was assessed between dexmedetomidine and a combination of dexmedetomidine-dexamethasone. Various meta-analyses were conducted demonstrating a significant reduction in pain score intraoperatively with a significant reduction in intraoperative fentanyl and postoperative analgesic requirement in the dexmedetomidine group compared to placebo [5,6,16]. The reduction of postoperative pain by dexmedetomidine could be explained by the activation of the α2-adrenoreceptor in the dorsal horn of the spinal cord, which inhibits the release of substance P, which modulates the transmission of nociceptive signals in the central nervous system, leading to reduction of nociceptive inputs during the acute postoperative period [20].

Intraoperative and postoperative vital parameters like heart rate, BP, and mean arterial pressure show persistently and significantly lower mean values in participants receiving dexmedetomidine (alone or combined with dexamethasone) than those receiving placebo. Although dexmedetomidine does lead to lower heart rate and BP compared to placebo, these effects mostly do not have significant consequences. Also, persistently stable cardiovascular parameters indicate better and adequate intra- and postoperative analgesia, sedation, and overall patient comfort.

Limitations

The adjuvant drugs were not taken into account in the analysis, and the time of day of the RSS assessment was not noted; the results must be interpreted accordingly.

## Conclusions

To conclude, vomiting may have varied adverse effects on patient recovery; administering dexmedetomidine in patients undergoing surgery under GA may help to reduce the incidence and severity of PONV, as well as provide adequate postoperative sedation and analgesia. Overall patient comfort is enhanced. We also conclude that dexmedetomidine may reduce PONV when administered alone as effectively as it does with dexamethasone.
